# Development of type 2 diabetes mellitus quality indicators in general practice by a modified Delphi method in Beijing, China

**DOI:** 10.1186/s12875-020-01215-9

**Published:** 2020-07-19

**Authors:** Guanghui Jin, Yun Wei, Yanli Liu, Feiyue Wang, Meirong Wang, Yali Zhao, Juan Du, Shuqi Cui, Xiaoqin Lu

**Affiliations:** 1 Department of General Practice, School of General Practice and Continuing Education, Capital Medical University, Beijing, People’s Republic of China; 2 Department of General Practice, Beijing Tiantan Hospital, Capital Medical University, Beijing, People’s Republic of China

**Keywords:** Quality of care, Quality indicators, Type 2 diabetes mellitus, Delphi method, General practice, Beijing

## Abstract

**Background:**

The service capacity of primary care has improved in China. General practice also takes growing responsibility in the management of type 2 diabetes mellitus, but there are concerns about the paucity of evidence of the quality of care delivered. And there is an absence of systematic quality indicators of type 2 diabetes mellitus in general practice in China. This study aimed to develop a set of type 2 diabetes mellitus quality indicators to facilitate quality measurement in general practice in China.

**Methods:**

Preliminary quality indicators were generated and refined by literature review and an expert consultation meeting. Two rounds of email-based Delphi survey and a consensus meeting were carried out to identify quality indicators. Delphi questionnaires with 43 indicators were sent to 30 participants in the first round. There were 16 general practitioners and 10 community health service center leaders from primary care, 3 endocrinologists and a primary care researcher in the first round. And 27 out of the 30 participants participated in the second round. The consensus meeting was held among 9 participants to refine the indicators and a last round of rating was carried out in the meeting. The indicators were rated in terms of importance and feasibility. The agreement criteria were defined as median ≥ 7.0 and ≥ 85.0% of ratings in the 7–9 tertile for importance; median ≥ 7.0 and ≥ 65.0, 70.0, 75.0% of ratings in the 7–9 tertile for feasibility respectively in the three rounds of rating.

**Results:**

After 2 rounds of Delphi survey and the consensus meeting, total 38 indicators achieved consensus for inclusion in the final set of indicators. The final set of indicators were grouped into 7 domains: access (5 indicators), monitoring (12 indicators), health counseling (7 indicators), records (2 indicators), health status (7 indicators), patient satisfaction (2 indicators) and self-management (3 indicators).

**Conclusions:**

A set of 38 potential quality indicators of type 2 diabetes mellitus in general practice were identified by an iterative Delphi process in Beijing, China. Preliminary approach for measurement and data collection were described. However, the indicators still need to be validated by testing in a further study.

## Background

General practice is an essential component of primary care providing high-quality care to individuals and families [1, 2]. In order to improve the accessibility and efficiency of the health care system, the government of China decided to strengthen primary care by establishing the community health service (CHS) system in 1997 [3]. Community health service institutions (CHSIs) are the main primary care institutions providing basic medical and public health services in China, which include community health service centers (CHSCs) and community health service stations (CHSSs) [4]. CHSCs usually comprise departments of general practice, traditional Chinese medicine, preventive care, rehabilitation, women health, laboratory tests, and pharmacy [4]. The CHSSs are affiliated institutions of CHSCs to cover areas distant away from CHSCs in the community [4]. CHSSs usually comprise departments of general practice and pharmacy. General practice is responsible for providing basic medical care in CHSIs. In China’s health care reform since 2009 [5], the government increased financial support to primary care institutions from 2.8 billion US dollars in 2008 to 20.3 billion US dollars in 2015 [6, 7]. The number of CHSIs increased from 22,656 in 2006 to 34,997 in 2018 [8]. There were 9352 CHSCs, 25,645 CHSSs and 156,800 registered general practitioners (GPs) in 2018 in China [8]. However, the choice of first contact of care is voluntary for the patients in China. General practices in CHSIs are often bypassed by the patients, who prefer to seek care from secondary and tertiary hospitals with better infrastructure and reputation [9]. The goal of the government is to establish a tiered health care system, in which primary care is the first contact of care collaborating with secondary and tertiary care hospitals [10]. Higher reimbursement rate and lower price of services were provided to incentivize patients to choose CHSIs as the first contact of care [10]. Visits to CHSIs increased from 484,516,000 in 2010 to 799,094,000 in 2018 [8]. Policy to develop general practice was also implemented, i.e. establishment of the nationwide general practice training system since 2011 [11]. GPs will be trained by a 3-year standard residency training program after medical school, with the goal of building up a workforce of 300,000 GPs nationwide till 2020 [11]. China has made remarkable progress in strengthening its primary care system. Nevertheless, the primary care system still faces challenges in workforce, incentive policies and quality of care (QOC) [12].

Chronic disease management is defined as a core task of general practice to tackle the prevalence of chronic diseases at the community level in China [11]. The prevalence of diabetes mellitus was 10.9% in China in 2013 [13]. It was the sixth leading cause of disability-adjusted life years (DALYs) in China in 2016 [14]. General practice has important roles in the management of type 2 diabetes mellitus (T2DM) [15, 16]. There has been a shift from hospital care to primary care in T2DM management in China [17]. General practice is responsible for the screening, long-term management and referral of T2DM patients in the community [18]. High quality care models e.g. patient centered medical home and chronic care model had been proven effective in improving diabetes care [19, 20]. China also launched a “family doctor contract service” model, in which the GPs will sign contracts with patients [21]. The model is intended to let patients have their personal doctors and improve the continuity of care [21]. A team approach is adopted in the model, with a GP, nurse and preventive care physician working together to provide comprehensive care for the patients [22, 23]. Electronic health record (EHR) systems are also developed in CHSIs. Basic modules in the EHR system comprise health profile, medical charts and check-up reports for T2DM management [24]. Innovations in information technologies such as electronic appointment system, distance monitoring and consultation were also piloted in CHSIs with better infrastructure [24, 25]. Despite introduction of the new model, there is little evidence on the quality of T2DM care in general practice in China [12]. One of the causes is the absence of systematic T2DM quality measures for general practice in China [12, 26].

QOC is an important area in primary care which draws international attention [27, 28]. Quality indicators (QIs) can be used to measure the quality of process, outcome and structure of health care [29, 30]. The QOC in general practice in China is mainly assessed by a pay for performance (PFP) scheme which contains 3 QIs on T2DM [31], including: (1) the ratio of T2DM patients being registered in the management of general practice; (2) the ratio of patients being followed up for at least 4 times per year; (3) the ratio of patients whose blood glucose is under control [31]. The GPs will be incentivized if they can achieve a predefined ratio for the QIs [31]. However, the indicators were not sufficient for reflecting the QOC in general practice [32]. Key QIs of clinical care are often unavailable, which impedes the generation of evidence of QOC in general practice [12]. Thus, scientific and feasible QIs are needed for the quality measurement in general practice in China. Many QIs are already available from different countries [33–35], however it should be prudent in the use of existing QIs because of the variation in professional culture and the complexity of clinical practice [36]. This study aimed to develop a set of QIs of T2DM in general practice by a modified Delphi method in Beijing to facilitate quality measurement in general practice in China.

## Methods

### The Delphi process

An iterative Delphi process was conducted to reach consensus on the indicators in this study. This technique was utilized because it had been shown to be an effective method for developing QIs in primary care research [37, 38]. The avoidance of face to face interaction among participants in Delphi survey can prevent individuals feeling intimidated and opinions can be expressed without pressure. However, the absence of face to face discussion prohibits the exchange of different perspectives [37]. Physical meeting can be held when reaching a consensus is difficult or to confirm the results of Delphi survey [39, 40].

**Fig. 1:**
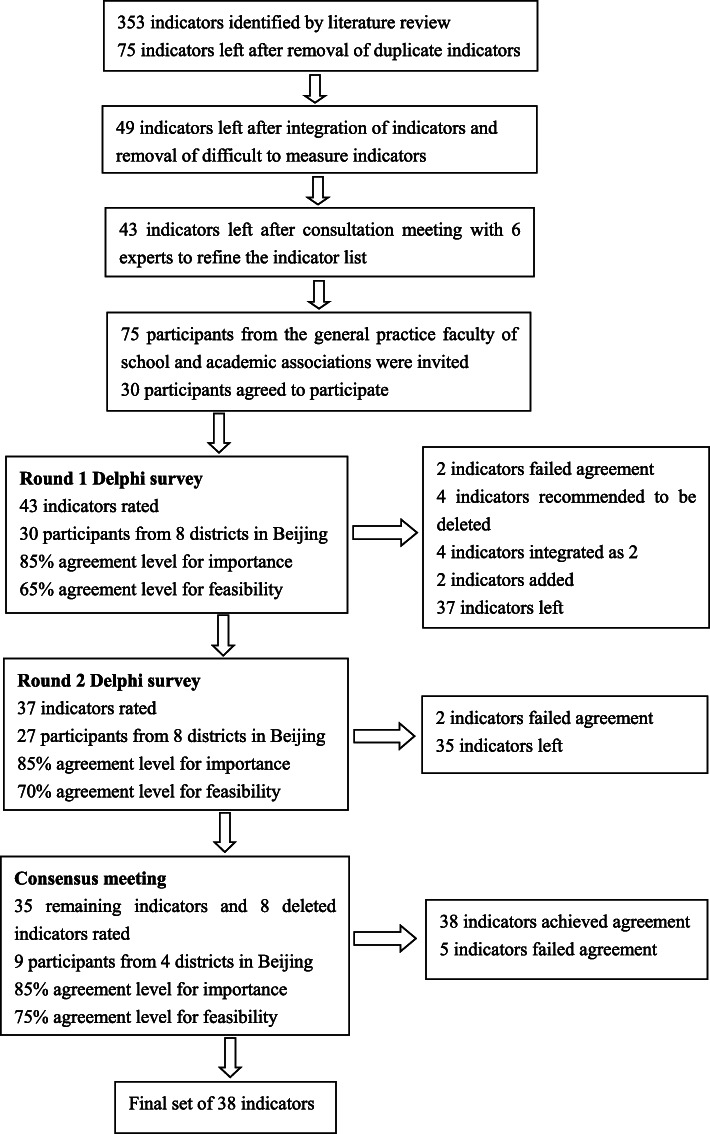
The process of the Delphi study. The Delphi process consisted of 2 rounds of survey and a consensus meeting. The list of indicators was modified after each round of rating. The consensus meeting involved discussion and the final rating process. 38 indicators were identified eventually

The whole Delphi process in this study consisted of: (1) literature review to screen potential indicators; (2) an expert consultation meeting to refine the preliminary indicators; (3) two rounds of email-based Delphi survey; (4) a face to face consensus meeting. The meeting was held because we perceived the necessity to confirm the results of Delphi survey and to refine the description of indicators. The study lasted from October 2018 to March 2019. Please see Fig. 1 for the process of the Delphi study.

### Panel of participants

A list of 75 potential participants was generated from the faculty of general practice of the school and members of general practice academic association who had active roles in general practice training and research in Beijing. The participants were professionals with different roles in T2DM management, including GPs, administrative leaders of CHSCs, endocrinologists in tertiary hospitals and primary care researchers. The participants were chosen based on the following criteria: (1) GP: working as first-line GP for over 5 years, teacher of GP training, experience in research projects and publications; (2) administrative leader of CHSC: working in the administration of CHSC for over 10 years, experience in quality measurement of T2DM care in general practice, teacher of GP training, experience in research projects and publications; (3) endocrinologist: working as endocrinologist for over 10 years, teacher of GP training, experience in research projects and publications; (4) primary care researcher: over 10 years of experience as primary care researcher, familiar with T2DM management, experience in QI development research. The participants were asked for their willingness to take part in the study. Thirty participants from Beijing who met the criteria agreed to take part in the study in the first round. Geographical distribution was also considered in selecting the participants. There are 16 districts in Beijing and the 30 participants covered 8 districts of Beijing.

The response rate in the second round was 90.0% (27/30). Three participants dropped out of the study due to the lack of availability. In the consensus meeting, 10 participants (1/3) were invited to represent the panel based upon their expertise in T2DM management, experience in quality measurement and availability to attend the meeting. Health professionals are promoted within a title system in terms of their work experience and research achievement in China [41]. There are junior grade, middle grade, associate senior grade and senior grade titles. Participants with relatively longer work experience and higher titles (associate senior grade and senior grade titles) were invited in the consensus meeting. Nine (9/10) participants attended the meeting. The primary care researcher who was a professor of university was invited but unavailable for the meeting, so the final indicators were sent to the researcher by email for further comments.

### Indicator selection and refinement

A preliminary list of indicators was constructed from three sources by literature review. Firstly, 2 published T2DM clinical guidelines from China and 4 guidelines from International Diabetes Federation (IDF), United States of America (USA), United Kingdom (UK) and Australia were reviewed [15, 18, 42–45]. Secondly, T2DM QIs produced by key organizations in China, USA, UK and Australia were reviewed [33, 34, 46, 47]. Thirdly, indicators were extracted from 73 published research papers on T2DM quality measurement in general practice which were identified by literature review. Please see Table 1 for the sources of clinical guidelines and indicators from key organizations.

**Table 1:** Sources of guidelines and indicators from key institutions reviewed

Type of sources	Sources	Institution	Publication year	Country
Clinical guidelines	Guidelines on the prevention and treatment of T2DM in China 2017 [42]	Chinese Medical Association, Diabetes Chapter	2017	China
	National guidelines on prevention and treatment of diabetes in primary care [18]	Chinese Medical Association, Diabetes Chapter	2018	China
	Standards of medical care in diabetes-2018 [45]	American Diabetes Association	2018	USA
	Type 2 diabetes in adults: management [44]	National Institute for Health and Care Excellence	2015	UK
	Global guideline for type 2 diabetes [43]	International Diabetes Federation	2005	Not applicable
	General practice management of type 2 diabetes 2016–18 [15]	The Royal Australian College of General Practitioners	2016	Australia
Indicators from key institutions	Community health service quality standard [46]	Community Health Association of China	2016	China
	Quality and outcomes framework [33]	National Health Service	2018	UK
	Primary care measures [34]	The Centers for Medicare & Medicaid Services	2016	USA
	Clinical indicators for Australian general practice [47]	The Royal Australian College of General Practitioners	2015	Australia

Abbreviation: *T2DM* Type 2 diabetes mellitus; *USA* United States of America; *UK* United Kingdom

Potential indicators were extracted from these sources and screened by a panel of 3 reviewers (JGH, WY, YLL) according to the following criteria: (1) the indicator was relevant to the management of T2DM in general practice; (2) the indicator had explicit standard or recommendation; (3) the indicator was measurable. When there were doubts about whether an indicator should be retained, the research team would discuss together to make a decision. Total 353 indicators were identified by the screening process. Duplicate indicators were deleted to form a preliminary list of 75 indicators. However, there were still indicators difficult to be measured and indicators needed to be integrated because of similar dimensions being measured. Thus, the indicators were discussed in detail one by one in a research team meeting. After further removal and integration, 49 indicators were left, which were categorized preliminarily into the clinical domains of T2DM management [35, 42].

Before constructing the Delphi questionnaire, we conducted a consultation meeting with six experts who had important roles in general practice training, quality measurement, and research in Beijing. There were 2 GPs, 1 director of CHSC and 3 primary care researchers. The GPs and director of CHSC were also teachers of general practice training in CHSCs and were experienced in quality measurement in general practice. The three primary care researchers were academic professors in the university with a lot of experience in general practice research. All the six experts also had active roles in general practice academic associations in China. The goals of the meeting were to: (1) refine the framework and description of the indicators; (2) recommend important indicators from the experts’ perspective; (3) identify indicators that should be further integrated; (4) decide the agreement criteria to be used in the Delphi rating process. After the meeting, a list of 43 indicators was finalized and categorized into nine domains.

### Delphi questionnaire

The indicators were developed into a Delphi questionnaire. Importance and feasibility were utilized as two dimensions to rate the indicators on a 1–9 Likert scale. Importance was defined as the extent to which the indicator was considered important for providing high quality T2DM care in general practice. Feasibility was defined as the extent to which the indicator could be feasibly used in the general practice setting. Spaces were left for the participants to make comments to the indicators or recommend new indicators. The materials of Delphi survey were sent to the participants by email including, the research background, instructions, and rating form. In the consensus meeting, rating forms were completed by the participants after discussion. Please see supplement file for the first-round rating form [see Additional file 1].

### Delphi survey

In the Delphi survey, the assumption was that most indicators would be considered as important, however the feasibility of indicators might be variable because of the variations in practice infrastructure and the complexity of real life practice. So, it was decided in the expert consultation meeting to set lower agreement level of ratings in feasibility than in importance and raise the agreement level between rounds, in case important indicators were deleted in early rounds. This approach has been adopted in previous Delphi research [38]. Please see Table 2 for the agreement criteria between rounds.

**Table 2:** Agreement criteria between Delphi rating rounds

Round of rating	Agreement of importance		Agreement of feasibility	
	Median	Percentage in 7–9 tertile	Median	Percentage in 7–9 tertile
Round 1	≥7.0	≥85.0%	≥7.0	≥65.0%
Round 2	≥7.0	≥85.0%	≥7.0	≥70.0%
Consensus meeting	≥7.0	≥85.0%	≥7.0	≥75.0%

In the first round, agreement criteria were defined as median ≥ 7.0 and ≥ 85.0% of ratings in the 7–9 tertile for importance and ≥ 65.0% of ratings in the 7–9 tertile for feasibility. Indicators recommended to be deleted by more than 3 participants would also be removed. The indicators were clarified and modified based on the rating results and feedback from the participants in the first round. The modified indicators were sent to the participants again for the second-round rating. The modifications and ratings in the first round were elaborated in a document with the second-round questionnaire. The participants were able to see their responses as well as the responses of the other participants without knowing each other’s identity. The agreement criteria were defined as median ≥ 7.0 and ≥ 85.0% of ratings in the 7–9 tertile for importance and ≥ 70.0% of ratings in the 7–9 tertile for feasibility in the second round.

In the consensus meeting, we presented the results of the first and second rounds of Delphi survey. The indicators deleted in the first and second rounds were discussed in detail again. The description of each indicator as well as the assumed approach for measurement and data collection were also clarified in the meeting. The remaining indicators and the deleted ones were then rated by the participants for the last time to confirm the results. The agreement criteria were defined as median ≥ 7.0 and ≥ 85.0% of ratings in the 7–9 tertile for importance and ≥ 75.0% of ratings in the 7–9 tertile for feasibility.

## Results

### Demographics of the participants in Delphi survey

The participants were from 8 districts in Beijing in the first and second rounds and 4 districts in the consensus meeting. There were more female participants in both rounds (70.0 and 66.7%) and the consensus meeting (77.8%). Over 50.0% of the participants were between 40 ~ 49 years old in both rounds (50.0 and 51.9%) and the consensus meeting (66.7%). Over half of the participants were GPs in both rounds (53.4 and 55.6%) and the consensus meeting (55.6%). Most of the participants had been working for 11 ~ 20 years in both rounds (56.7 and 59.3%) and the consensus meeting (77.8%). In the first and second rounds, 46.7 and 48.2% of the participants had master’s degree. And 55.6% of the participants had master’s degree in the consensus meeting. In the first and second rounds, 33.3 and 29.6% of the participants were with senior grade title. In the consensus meeting, 55.6% of the participants were with senior grade title. Please see Table 3.

**Table 3:** Demographics of the participants

Demographics	Round 1	Round 2	Consensus meeting
Gender			
Male	9 (30.0%)	9 (33.3%)	2 (22.2%)
Female	21 (70.0%)	18 (66.7%)	7 (77.8%)
Age			
30 ~ 39	9 (30.0%)	8 (29.6%)	1 (11.1%)
40 ~ 49	15 (50.0%)	14 (51.9%)	6 (66.7%)
50~	6 (20.0%)	5 (18.5%)	2 (22.2%)
Professional field			
GP	16 (53.4%)	15 (55.6%)	5 (55.6%)
Leader of CHSI	10 (33.3%)	8 (29.6%)	3 (33.3%)
Endocrinologist	3 (10.0%)	3 (11.1%)	1 (11.1%)
Primary care researcher	1 (3.3%)	1 (3.7%)	0
Work year			
5 ~ 10	7 (23.3%)	6 (22.2%)	1 (11.1%)
11 ~ 20	17 (56.7%)	16 (59.3%)	7 (77.8%)
21 ~ 30	6 (20.0%)	5 (18.5%)	1 (11.1%)
Highest degree			
PhD	2 (6.6%)	2 (7.4%)	0
Master	14 (46.7%)	13 (48.2%)	5 (55.6%)
Bachelor	14 (46.7%)	12 (44.4%)	4 (44.4%)
Professional title^a^			
Senior grade title	10 (33.3%)	8 (29.6%)	5 (55.6%)
Associate senior grade title	12 (40.0%)	12 (44.4%)	3 (33.3%)
Middle grade title	8 (26.7%)	7 (26.0%)	1 (11.1%)

Abbreviation: *GP* General practitioner; *CHSI* Community health service institution

Note: ^a^ professional titles include junior grade, middle grade, associate senior grade and senior grade titles, which are based upon work experience and research achievement of health professionals

### Delphi survey and consensus meeting

In the first round, all the 43 indicators achieved 85.0% agreement except one indicator “admission days” (83.3%) in terms of importance. In terms of feasibility, one indicator “ankle-brachial index monitoring”(63.3%)failed to achieve 65.0% agreement. Four indicators were recommended to be removed by more than 3 participants, despite the agreement they had achieved. So, 6 indicators were deleted in the first round. Four indicators were integrated as two indicators, and 2 new indicators were added. There were 37 indicators left after the first round.

In the second round, all the 37 indicators achieved 85.0% agreement in terms of importance. In terms of feasibility, two indicators, including “psychological counseling” (59.3%) and “quality of life” (66.7%) failed to achieve 70.0% agreement and were deleted. There were 35 indicators left after the second round.

In the third round, 43 indicators including the deleted ones were rated after discussion. And 42 indicators achieved 85.0% agreement for importance. In terms of feasibility, 38 indicators achieved 75.0% agreement. Five indicators still failed to achieve agreement. Three indicators deleted in the first 2 rounds of Delphi survey were resurrected and modified in the consensus meeting. They were “psychological assessment or counseling”, “hypoglycemia episodes” and “T2DM related admissions to hospitals”. Please see Table 4 for the distribution of indicators by agreement level and Table 5 for the five deleted indicators. Modifications on the list of indicators in each round were presented in Fig. 1. The results of ratings in the Delphi survey and the consensus meeting were provided in an additional file [see Additional file 2].

**Table 4:** Distribution of indicators by agreement level

Agreement level	Round 1		Round 2		Consensus meeting	
	Importance	Feasibility	Importance	Feasibility	Importance	Feasibility
85.0%~	42	16	37	25	42	35
75.0%~	1	15	0	5	0	3
70.0%~	0	9	0	5	0	0
65.0%~	0	2	0	1	1	3
< 65.0%	0	1	0	1	0	2
Total	43	43	37	37	43	43

**Table 5:** Five deleted indicators and ratings in the consensus meeting

Indicator	Importance		Feasibility		Comments
	Median	Agreement	Median	Agreement	
Ankle-brachial index monitoring	9.0	88.9%	6.5	44.4%	The test is not available in many CHSIs
T2DM related admission days in hospital	9.0	100.0%	7.0	66.7%	Lack of access to hospital EHR and difficulty to obtain from patient survey
Rational use of medicines	9.0	88.9%	7.0	66.7%	Difficult to define and difficulty in data collection in the current EHR
Incidence of complications	9.0	88.9%	7.0	66.7%	Many confounding factors and the need of long-term observation
Quality of life	8.0	66.7%	6.0	33.3%	Many confounding factors on the indicator

Abbreviations: *T2DM* Type 2 diabetes mellitus; *CHSI* Community health service institution; *EHR* Electronic health record

### The final set of indicators

After 2 rounds of Delphi survey and the consensus meeting, a total of 38 indicators achieved consensus for inclusion in the final set of indicators. The 38 indicators were grouped into 7 domains: access (5 indicators), monitoring (12 indicators), health counseling (7 indicators), records (2 indicators), health status (7 indicators), patient satisfaction (2 indicators) and self-management (3 indicators). Potential data sources and measurement were also recommended by the panel of participants. Please see Table 6 for the final set of indicators.

**Table 6:** Description of the final set of indicators

Categories	Indicators	Description	Measurement	Data source
1. Access	1.1 Personal doctor	GP is the personal doctor providing continuous care for T2DM patient	The percentage of T2DM patients who recognize that they are being managed by a personal doctor	Patient survey
	1.2 GP team	Patient is being managed by a functioning GP team (a GP, nurse, and preventive care physician)	The percentage of T2DM patients who recognize that they are being managed by a functioning GP team	Patient survey
	1.3 Waiting time	Waiting time for consultation is reasonable for the patient	The percentage of T2DM patients who recognize that the waiting time for consultation is reasonable	Patient survey
	1.4 Health advice	Seeking health advice from the GP team is convenient for the patient	The percentage of T2DM patients who recognize that it is convenient to seek health advice from the GP team	Patient survey
	1.5 Referral access	Patient has ensured referral access to necessary specialist care	The percentage of T2DM patients who recognize that referral access to necessary specialist care is ensured	Patient survey
2. Monitoring	2.1 Regular follow up	At least 4 times of follow up by the GP team in the audit year	The percentage of T2DM patients who are followed up for at least 4 times by the GP team in the audit year	EHR
	2.2 Plasma blood glucose monitoring	At least 4 measurements of plasma blood glucose test (fasting or post-prandial) by the GP team in the audit year	The percentage of T2DM patients who have at least 4 measurements of plasma blood glucose test (fasting or post-prandial) by the GP team in the audit year	EHR
	2.3 HbA1c monitoring	At least 2 measurements of HbA1c test in the audit year	The percentage of T2DM patients who have at least 2 measurements of HbA1c test in the audit year	EHR
	2.4 BP monitoring	At least 4 measurements of BP by the GP team in the audit year	The percentage of T2DM patients who have at least 4 measurements of BP by the GP team in the audit year	EHR
	2.5 Lipid monitoring	At least 1 measurement of lipid test in the audit year	The percentage of T2DM patients who have at least 1 measurement of lipid test in the audit year Tests: TC, TG, LDL-C, HDL-C	EHR
	2.6 BMI monitoring	At least 1 measurement of BMI in the audit year	The percentage of T2DM patients who have at least 1 measurement of BMI in the audit year	EHR
	2.7 Waist circumference monitoring	At least 1 measurement of waist circumference in the audit year	The percentage of T2DM patients who have at least 1 measurement of waist circumference in the audit year	EHR
	2.8 ECG monitoring	At least 1 measurement of ECG in the audit year	The percentage of T2DM patients who have at least 1 measurement of ECG in the audit year	EHR
	2.9 Nephropathy monitoring	At least 1 nephropathy examination in the audit year	The percentage of T2DM patients who have at least 1 nephropathy examination in the audit year Tests: creatinine, blood urea nitrogen, urine protein	EHR
	2.10 Retinopathy monitoring	At least 1 retinopathy examination in the audit year	The percentage of T2DM patients who have at least 1 retinopathy examination in the audit year Tests: vision, funduscope or fundus photography	EHR
	2.11 Peripheral neuropathy monitoring	At least 1 peripheral neuropathy examination in the audit year	The percentage of T2DM patients who have at least 1 peripheral neuropathy examination in the audit year Tests: temperature, pinprick sensation and vibration sensation, 10-g monofilament testing	EHR
	2.12 Foot monitoring	At least 1 diabetic foot examination in the audit year	The percentage of T2DM patients who have at least 1 diabetic foot examination in the audit year Tests: Skin inspection, pulse palpation	EHR
3. Health counseling	3.1 Diet counseling	Diet counseling is provided for the patient in the audit year	The percentage of T2DM patients who are provided with diet counseling in the audit year	Patient survey
	3.2 Exercise counseling	Exercise counseling is provided for the patient in the audit year	The percentage of T2DM patients who are provided with exercise counseling in the audit year	Patient survey
	3.3 Psychological assessment or counseling	Psychological assessment is provided for the patient or referral to professional counseling when necessary in the audit year	The percentage of T2DM patients who are provided with psychological assessment or referral to professional counseling when necessary in the audit year	Patient survey
	3.4 Smoking assessment or counseling	Smoking assessment is provided for the patient or referral to professional counseling when necessary in the audit year	The percentage of T2DM patients who are provided with smoking assessment or referral to professional counseling when necessary in the audit year	Patient survey
	3.5 Hypoglycemia awareness counseling	Hypoglycemia awareness counseling is provided for the patient in the audit year	The percentage of T2DM patients who are provided with hypoglycemia awareness counseling in the audit year	Patient survey
	3.6 Medication safety counseling	Medication safety counseling is provided for the patient in the audit year	The percentage of T2DM patients who are provided with medication safety counseling in the audit year	Patient survey
	3.7 Emergency help counseling	Emergency help counseling is provided for the patient to improve the patient’s knowledge on seeking emergency help in the audit year	The percentage of T2DM patients who are provided with emergency help counseling in the audit year	Patient survey
4. Records	4.1 Follow up records	Follow up records are kept in the audit year	The percentage of T2DM patients whose follow up records are kept in the audit year	EHR
	4.2 Annual management summary report	Annual management summary report, including summary of management status and comprehensive physical examination report are kept in the audit year	The percentage of T2DM patients whose annual management summary reports are kept in the audit year	EHR
5. Health status	5.1 Blood glucose target	The patient’s latest fasting blood glucose is between 4.4–7.0 mmol/L; and post prandial blood glucose < 10.0 mmol/L in the audit year	The percentage of T2DM patients whose latest blood glucose is under control in the audit year based on the following criteria: Fasting blood glucose is between 4.4–7.0 mmol/L; Post prandial blood glucose < 10.0 mmol/L.	EHR
	5.2 HbA1c target	The patient’s latest HbA1c < 7%; or HbA1c < 8% (for patient with severe hypoglycemia or age ≥ 80 or micro or macro vascular complications) in the audit year	The percentage of T2DM patients whose latest HbA1c is under control in the audit year based on the following criteria: HbA1c < 7%; or HbA1c < 8% (for patients with severe hypoglycemia or age ≥ 80 or micro or macro vascular complications).	EHR
	5.3 BP target	The patient’s latest BP < 130/80 mmHg; or BP < 140/90 mmHg for old patient or patient with CHD in the audit year	The percentage of T2DM patients whose latest BP is under control in the audit year based on the following criteria: BP < 130/80 mmHg; or < 140/90 for old patient or patient with CHD	EHR
	5.4 Blood lipid target	The patient’s latest LDL-C < 1.8 mmol/L (for patient with ASCVD); or LDL-C < 2.6 mmol/L (for patient without ASCVD) in the audit year	The percentage of T2DM patients whose latest LDL-C is under control in the audit year based on the following criteria: LDL-C < 1.8 mmol/L (with ASCVD); or LDL-C < 2.6 mmol/L (without ASCVD).	EHR
	5.5 BMI target	The patient’s latest BMI < 24 kg/m^2^ in the audit year	The percentage of T2DM patients whose latest BMI < 24 kg/m^2^ in the audit year	EHR
	5.6 Hypoglycemia episodes	The patient’s episodes of hypoglycemia (including symptomatic hypoglycemia and test results) in the most recent month during the audit year	The percentage of T2DM patients who experienced episodes of hypoglycemia (including symptomatic hypoglycemia and test results) in the most recent month during the audit year	Patient survey
	5.7 T2DM related admissions to hospital	The patient’s T2DM related admissions to hospital in the audit year	The percentage of T2DM patients who experienced T2DM related admissions to hospital in the audit year	Patient survey
6. Patient satisfaction	6.1 Satisfaction with treatment	The patient’s perceived satisfaction with treatment	Score of DTSQ from T2DM patients in the audit year	Patient survey
	6.2 Satisfaction with communication	The patient’s perceived satisfaction with communication	Score of communication satisfaction from T2DM patients in the audit year	Patient survey
7. Self-management	7.1 Knowledge of self-management	The patient’s knowledge of self-management	Score of MDKT from T2DM patients in the audit year	Patient survey
	7.2 Adherence to medication	The patient’s adherence to medication	Score of Morisky 8 Item Scale from T2DM patients in the audit year	Patient survey
	7.3 Adherence to healthy behavior	The patient’s adherence to healthy behavior	Score of SDSCA from T2DM patients in the audit year	Patient survey

Abbreviations: *GP* General practitioner; *T2DM* Type 2 diabetes mellitus; *EHR* Electronic health record; *HbA1c* Glycosylated hemoglobin; *BP* Blood pressure; *TC* Total cholesterol; *TG* Triglyceride; *LDL-C* Low density lipoprotein cholesterol; *HDL-C* High density lipoprotein cholesterol; *BMI* Body mass index; *ECG* Electrocardiogram; *CHD* Coronary heart disease; *ASCVD* Arteriosclerotic cardiovascular disease; *DTSQ* Diabetes Treatment Satisfaction Questionnaire; *MDKT* Michigan Diabetes Knowledge Test; *SDSCA* Summary of Diabetes Self-Care Activities Measure

## Discussion

QOC measurement of T2DM in general practice has been widely carried out in many countries both in national programs and individual studies [33–35]. The Quality and Outcomes Framework (QOF) is an extensive PFP program in general practice in the UK and there were 11 QIs of T2DM in the QOF (2018/2019) [33]. A systematic review showed the number of diabetes QIs in primary care research varied widely from 3 to 57, with a median of 14 indicators [35]. Despite extensive international experiences in QOC measurement of T2DM in general practice, little evidence on the QOC of T2DM in general practice is available in China [12, 26]. The current 3 QIs of T2DM in the government-funded PFP program in general practice has been used for nearly a decade [31], however important indicators related to risk factors such as glycosylated hemoglobin (HbA1c) and low-density lipoprotein cholesterol (LDL-C) are still missing. The EHR system was also under-utilized for quality measurement in the PFP program [12]. A systematic review in Asia and the Middle East showed the paucity of research from these areas on the quality of diabetes care in primary care [26]. The absence of systematic QIs might be one of the causes for the lack of QOC studies in general practice in China [12, 26].

Unlike recommendations in clinical guidelines, feasibility is a critical prerequisite for QIs [37, 48]. The translation of recommendations into operationalizable QIs in general practice requires rigorous process [35, 37]. Thus, the aim of this study was to identify indicators that were both important and feasible in the general practice context in China. A set of 38 potential indicators to reflect the QOC of T2DM in general practice were identified in this study based on an iterative process including literature review, consultation meeting, Delphi survey and consensus meeting. Seven domains of indicators were identified, including access, monitoring, health counseling, records, health status, patient satisfaction and self-management. Most indicators in monitoring, health counseling and health status were process and intermediate outcome indicators derived from clinical guidelines which were frequently used in previous T2DM quality measurement studies [35, 49]. Individualized targets had been recommended for intermediate outcome indicators including HbA1c, blood pressure (BP), LDL-C in both Chinese and international clinical guidelines [42, 45], however using individualized targets in quality measurement is still a complex problem to be solved [50]. Considering the feasibility of data collection and the recommended values in Chinese clinical guidelines [18, 42], we used different HbA1c, BP, LDL-C targets for different patient groups in this study instead of setting one value for all patient groups. Patient safety is an important aspect of T2DM care, which is emphasized by recent clinical guidelines [45]. However, few indicators related to patient safety are available in most QIs used previously [35, 51]. We made an attempt to identify indicators in this domain on the basis of literature review and the participants’ consensus. Three indicators including “hypoglycemia awareness counseling”, “medication safety counseling”, and “emergency help counseling” were identified as important patient safety indicators in the health counseling domain. And “hypoglycemia episodes” and “T2DM related admissions to hospital” in the health status domain were identified. These indicators have not been frequently used in previous studies [35, 51], however the participants agreed on the practical importance of the indicators in real life practice.

High quality T2DM care has shifted to a patient centered approach [15, 45]. There were calls for patient centered indicators as well [50, 52]. We tried to incorporate indicators to measure domains of patient centered care, i.e. access of care, self-management and patient satisfaction (patient experience) [52, 53]. Access of care is a fundamental feature of general practice [1, 53], however it is difficult to translate the concept into measurable indicators. The Primary Care Assessment Tool (PCAT) had been used for assessing dimensions of access in primary care in China [54]. In this study, we identified 5 indicators based on the access dimensions of PCAT and the participants’ opinions. There were having a personal doctor for T2DM patients, patients’ perception of a functional GP team, waiting time, access to seeking health advice, and referral access to necessary specialist care. These indicators were also relevant to the essential medical services provided in general practice of CHSIs in China [21]. Patient self-management is critical for the outcome of T2DM care and is proposed as a QI to assess how patients are doing in T2DM management [50]. We identified 3 self-management indicators, i.e. knowledge of self-management, adherence to medication and adherence to healthy behavior in this study. Quality measures in adherence may facilitate new efforts to improve adherence and patient outcomes [50], however controversy exists in using adherence indicators due to the difficulty in measurement. There are scales in Chinese version with proved validity and reliability to measure T2DM health literacy and adherence [55–57]. We proposed the Michigan Diabetes Knowledge Test (MDKT) for self-management knowledge [58], Morisky 8 Item Scale for medication adherence [59], and the Summary of Diabetes Self-Care Activities Measure (SDSCA) for healthy behavior adherence as preliminary tools for the measurement of the indicators [60]. However, the feasibility of this approach needs to be verified by testing the indicators. Patient satisfaction had been used as an indicator in previous T2DM quality measurement studies [61]. The patient’s satisfaction with treatment and communication were identified as patient experience indicators in this study. And the Diabetes Treatment Satisfaction Questionnaire (DTSQ) was proposed for measuring the patient’s satisfaction with treatment [61]. Five indicators failed to meet the agreement criteria, because the indicators were difficult to be defined or measured in the current general practice context of China. For example, the participants agreed that “rational use of medicines” was important, however it was difficult to achieve consensus on how the indicator could be properly defined or feasibly assessed via the current EHR system in China.

The indicators in this study were recommended to be measured on the accumulation of individual patient data by two methods: EHR review and patient survey. Indicators in monitoring, records and health status were recommended to be measured via the EHR system. EHR systems have been developed in CHSIs in the health care reform in China [21]. The growing use of EHR systems in CHSIs provides an opportunity to assess the comprehensive QOC of T2DM in general practice [25, 62]. However, there are still problems in the current EHR systems in CHSIs. Because different systems are being used among CHSIs [25, 62], the modules of clinical quality information among different systems might be fragmented, which would increase the difficulty of data extraction. Partial clinical indicators might even be unavailable in CHSIs with less-developed EHR systems [12]. A widely used and accurate EHR system for data collection needs to be developed for general practice in China. The clinical QIs can be embedded into the EHR system to improve the quality of clinical information, which might also be helpful for the decision making based on systematic and timely clinical information [12, 63]. Nurses in the GP team also have growing roles in terms of health education and routine data collection of T2DM follow up in CHSIs [25]. This might also facilitate the data extraction for quality measurement from the EHR system. Indicators in access, health counseling, patient satisfaction and self-management were recommended to be measured by patient survey. Self-report measures have been found to be feasible QIs in primary care settings [52]. Information technologies such as web-based survey methods can improve the efficiency of patient survey [64].

This Delphi study provided a basis for the QOC measurement of T2DM in general practice in China. However, the indicators still need to be tested in real life practice in a further study [37, 48]. Critical aspects need to be assessed in testing the indicators i.e. data availability in the current EHR system in different CHSIs, accuracy of data, work load of data collection, reliability and reproducibility of data extraction, acceptability of the indicators for GPs being measured and stakeholders in CHSIs, and responsiveness to the change of QOC of T2DM [37, 48]. Composite QIs had been widely adopted in quality measurement [65, 66]. Approaches can be used to combine indicators to assess the overall QOC of T2DM, which enables comparison or ranking among general practices [67]. However, there are problems in using composite indicators, e.g. availability of data for the indicators among different CHSIs, bias caused by missing values, appropriate weight for the indicators and uncertainty in the final score [50, 68]. Thus, it should be prudent to use the set of indicators as composite indicators. Scoring strategies should use weights based on clinical importance and balance the weights to the dynamic changes of QOC in general practice [50, 68]. Detailed individual indicator data should be provided for the clear instruction on data collection, missing data processing and the scoring protocol. Case-mix adjustments for the indicator might be performed with detailed patient level information [50, 68].

### Limitation of the study

There are limitations of this study. The study was conducted in Beijing, all the participants were health professionals from Beijing. So, the application of the indicators in other regions should be based on the local health policy and available health resources. However, CHSIs are set up in accordance with standard specifications from the government [4], and over half of the participants were GPs and administrative leaders from CHSIs, which may improve the feasibility of the indicators in general practice setting. QOC in T2DM could encompass very broad dimensions. Some aspects of T2DM care might not be reflected in the indicators of this study, however, we attempted to identify the indicators considered both important and feasible in the current general practice context in China. Patients have more and more important roles in the development of QIs [52], but no T2DM patient was involved in this study. This might impede the opportunity to identify important indicators from the patient’s perspective. Further study should be done to explore the patient’s perspective on the QOC of T2DM in general practice in China.

## Conclusions

In summary, we identified a set of 38 potential QIs on T2DM care in general practice by an iterative Delphi process in Beijing, China. Preliminary approach for measurement and data collection of the QIs were recommended. However, the indicators still need to be validated by testing in real life practice in a further study.

## Supplementary information

**Additional file 1.** Rating form in the first round-translation

**Additional file 2.** Results of ratings in the Delphi survey and consensus meeting

## Data Availability

The datasets used and/or analyzed during the current study are available from the corresponding author (xq6518@163.com) on reasonable request.

## Abbreviations

CHS: Community health service
CHSI: Community health service institution
CHSC: Community health service center
CHSS: Community health service station
GP: General practitioner
QOC: Quality of care
DALYs: Disability-adjusted life years
T2DM: Type 2 diabetes mellitus
EHR: Electronic health record
QI: Quality indicator
PFP: Pay for performance
IDF: International diabetes federation
USA: United States of America
UK: United Kingdom
HbA1c: Glycosylated hemoglobin
BP: Blood pressure
TC: Total cholesterol
TG: Triglyceride
LDL-C: Low density lipoprotein cholesterol
HDL-C: High density lipoprotein cholesterol
BMI: Body mass index
ECG: Electrocardiogram
CHD: Coronary heart disease
ASCVD: Arteriosclerotic cardiovascular disease
DTSQ: Diabetes treatment satisfaction questionnaire
MDKT: Michigan diabetes knowledge test
SDSCA: Summary of diabetes self-care activities measure
QOF: Quality and Outcomes Framework

## References

[1] Kidd M. The contribution of family medicine to improving health systems: a guidebook from the World Organization of Family Doctors. 2nd ed. London: Radcliffe Publishing Ltd; 2013.

[2] Kringos DS, Boerma W, Hutchinson A, Der Zee JV, Groenewegen PP (2010). The breadth of primary care: a systematic literature review of its core dimensions. BMC Health Serv Res.

[3] The State Council of China. The decision on health care reform and development [in Chinese]. 1997. http://www.nhc.gov.cn/wjw/zcjd/201304/743ba60a223646cd9eb4441b6d5d29fa.shtml. Accessed 1 May 2020.

[4] Ministry of Health of China. Regulations on community health service institutions [in Chinese]. 2006. http://www.gov.cn/zwgk/2006-08/10/content_359147.htm. Accessed 1 May 2020.

[5] The State Council of China. Guidelines on deepening the health care reform [in Chinese]. 2009. http://www.gov.cn/gongbao/content/2009/content_1284372.htm. Accessed 1 May 2020.

[6] Ministry of Health of China. China health statistical yearbook 2009 [in Chinese]. Beijing: Peking Union Medical College Publishing House; 2010.

[7] National Health and Family Planning Commission of China. China health and family planning statistical yearbook 2016 [in Chinese]. Beijing: Peking Union Medical College Publishing House; 2017.

[8] National Health Commission of China. China health statistical yearbook 2019 [in Chinese]. Beijing: Peking Union Medical College Publishing House; 2019.

[9] Shao S, Zhao FF, Wang J, Feng L, Lu XQ, Du J, et al. The ecology of medical care in Beijing. Plos One. 2013;8(12): e82446. 10.1371/journal.pone.0082446 PMC3855438 24340029

[10] The State Council of China. Guidelines on facilitating the establishment of tiered health care system [in Chinese]. 2015. http://www.gov.cn/zhengce/content/2015-09/11/content_10158.htm. Accessed 1 May 2020.

[11] The State Council of China. Guidelines on establishing general practice system. [in Chinese] 2011. http://www.gov.cn/zwgk/2011-07/07/content_1901099.htm. Accessed 1 May 2020.

[12] Li X, Lu J, Hu S, Cheng KK, De Maeseneer J, Meng Q (2017). The primary health-care system in China. Lancet.

[13] Wang L, Gao P, Zhang M, Huang Z, Zhang D, Deng Q (2017). Prevalence and ethnic pattern of diabetes and prediabetes in China in 2013. JAMA.

[14] Liu M, Liu S, Wang L, Bai YM, Zeng X, Guo HB (2019). Burden of diabetes, hyperglycaemia in China from to 2016: findings from the 1990 to 2016, global burden of disease study. Diabetes Metab.

[15] Royal Australian College of General Practitioners. General practice management of type 2 diabetes 2016–18. 2016. https://www.racgp.org.au/clinical-resources/clinical-guidelines/key-racgp-guidelines/management-of-type-2-diabetes. Accessed 1 May 2020.

[16] John GC, Tor C, Anne KJ, Geir T, Marie FH, Wibeche I (2009). Quality of care for patients with type 2 diabetes in primary care in Norway is improving: results of cross-sectional surveys of 33 general practices in 1995 and 2005. Diabetes Care.

[17] The State Council of China. Long term plan (2017–2025) for prevention and treatment of chronic diseases in China. 2017. http://www.gov.cn/xinwen/2017-02/14/content_5167942.htm. Accessed 1 May 2020.

[18] Chinese Diabetes Mellitus Association (2018). National guidelines for the prevention and control of diabetes in primary care (2018) [in Chinese]. Chin J Intern Med.

[19] Friedberg MW, Rosenthal MB, Werner RM, Volpp KG, Schneider EC (2015). Effects of a medical home and shared savings intervention on quality and utilization of care. JAMA Intern Med.

[20] Stellefson M, Dipnarine K, Stopka C (2013). The chronic care model and diabetes management in US primary care settings: a systematic review. Prev Chronic Dis.

[21] National Health and Family Planning Commission of China. Guidelines on facilitating the family doctor contract service [in Chinese]. 2016. http://www.gov.cn/xinwen/2016-06/06/content_5079984.htm. Accessed 1 May 2020.

[22] Wei XJ, Wu H, Yu HY, Cui SQ, Ge CY, Jia HY (2017). Role of family doctor service team in community-based integrated management of diabetes [in Chinese]. Chi Gen Prac..

[23] Chen SR, Wang L (2018). Evaluation of the effect of family doctor responsibility contract service on community type 2 diabetes mellitus intervention [in Chinese]. Chi Prim Heal Care.

[24] Wei XJ, Wu H, Yu HY, Ge CY, Jia HY, Wang L (2017). Practical effect of family doctor service on promoting hierarchical diagnosis and treatment in Fangzhuang community [in Chinese]. Chi Gen Prac.

[25] Wang Q, Li AL, Yi M, Dong T, Li QX, Liu XL (2019). Application of diabetes management module in electronic contracted family doctor services management platform:a survey in Wuhou District [in Chinese]. Chi Gen Prac.

[26] Shivashankar R, Kirk K, Kim WC, Rouse C, Tandon N, Narayan KM (2015). Quality of diabetes care in low- and middle-income Asian and middle eastern countries (1993-2012): 20-year systematic review. Diabetes Res Clin Pract.

[27] Institute of Medicine (2001). Crossing the quality chasm: a new health system for the 21st century.

[28] World Organization of Family Doctors. Statement of principles of the WONCA Working Party on Quality and Safety in Family Medicine. 2013. https://www.globalfamilydoctor.com/groups/WorkingParties/QualitySafety.aspx. Accessed 1 May 2020.

[29] Nicolucci A, Greenfield S, Mattke S. Selecting indicators for the quality of diabetes care at the health systems level in OECD countries. Int J Qual Health Care. 2006:26–30. 10.1093/intqhc/mzl023 16954513

[30] American Academy of Family Physicians. What are the types of measures?. 2020. https://www.aafp.org/practice-management/improvement/measures.html. Accessed 1 May 2020.

[31] Ministry of Health of China. Pay for performance in community health service institutions [in Chinese]. 2011. http://www.gov.cn/gzdt/2011-06/30/content_1896720.htm. Accessed 1 May 2020.

[32] Peng YC, Su N, Chen Q, He YJ, Liang WN (2011). Evaluation of current performance appraisal system by community health service staff [in Chinese]. Chi Gen Prac.

[33] National Health Service. Quality and outcomes framework 2018/19. 2019. https://digital.nhs.uk/data-and-information/publications/statistical/quality-and-outcomes-framework-achievement-prevalence-and-exceptions-data/2018-19-pas. Accessed 1 May 2020.

[34] Centers for Medicare & Medicaid Services. Primary care measures. 2016. https://www.cms.gov/Medicare/Quality-Initiatives-Patient-Assessment-Instruments/QualityMeasures/Core-Measures.html. Accessed 1 May 2020.

[35] Calsbeek H, Ketelaar NA, Faber MJ, Wensing M, Braspenning JC (2013). Performance measurements in diabetes care: the complex task of selecting quality indicators. Int J Qual Health Care.

[36] Marshall M, Shekelle PG, Mcglynn EA, Campbell S, Brook RH, Roland M (2003). Can health care quality indicators be transferred between countries. Qual Saf Health Care.

[37] Campbell S, Braspenning JC, Hutchinson A, Marshall M (2002). Research methods used in developing and applying quality indicators in primary care. Qual Saf Health Care.

[38] Shield T, Campbell S, Rogers A, Worrall A, Chewgraham C, Gask L (2003). Quality indicators for primary care mental health services. Qual Saf Health Care.

[39] Boulkedid R, Abdoul H, Loustau M, Sibony O, Alberti C. Using and reporting the Delphi method for selecting healthcare quality indicators: a systematic review. Plos One. 2011;6(6):e20476. 10.1371/journal.pone.0020476 PMC3111406 21694759

[40] Albarqouni L, Hoffmann T, Straus S, Olsen N, Young T, Ilic D (2018). Core competencies in evidence-based practice for health professionals: consensus statement based on a systematic review and Delphi survey. JAMA Netw Open.

[41] Ministry of Health of China. Guidelines on building up urban community health service workforce [in Chinese]. 2006. http://www.gov.cn/zwgk/2006-08/10/content_359177.htm. Accessed 1 May 2020.

[42] Chinese Diabetes Mellitus Association (2018). Guidelines on the prevention and treatment of type 2 diabetes mellitus in China 2017 [in Chinese]. Chin J Diabetes Mellitus.

[43] International Diabetes Federation. Global guideline for type 2 diabetes. 2012. https://www.idf.org/e-library/guidelines/79-global-guideline-for-type-2-diabetes.html. Accessed 1 May 2020.

[44] National Institute for Health and Care Excellence. Type 2 diabetes in adults: management. 2015. https://www.nice.org.uk/guidance/ng28. Accessed 1 May 2020. 26741015

[45] American Diabetes Association (2018). Standards of medical care in diabetes-2018. Diabetes Care.

[46] Community Health Association of China. Community health service quality standards [in Chinese]. 2016. http://www.chs.org.cn/news/show.php?itemid=60. Accessed 1 May 2020.

[47] The Royal Australian College of General Practitioners. Clinical indicators for Australian general practice. 2015. https://www.racgp.org.au/running-a-practice/practice-management/general-practice-governance/clinical-indicators. Accessed 1 May 2020.

[48] Campbell S, Kontopantelis E, Hannon K, Burke MJ, Barber A, Lester H (2011). Framework and indicator testing protocol for developing and piloting quality indicators for the UK quality and outcomes framework. BMC Fam Pract.

[49] Seddon ME, Marshall M, Campbell S, Roland M (2001). Systematic review of studies of quality of clinical care in general practice in the UK, Australia and New Zealand. BMJ Qual Saf.

[50] Oconnor PJ, Bodkin NL, Fradkin JE, Glasgow RE, Greenfield S, Gregg EW (2011). Diabetes performance measures: current status and future directions. Diabetes Care.

[51] Rodriguezgutierrez R, Lipska KJ, Mccoy RG, Ospina NS, Ting HH, Montori VM (2016). Hypoglycemia as an indicator of good diabetes care. BMJ.

[52] Russell EG, Malinda P, Soren ES (2008). Where is the patient in diabetes performance measures?. Diabetes Care.

[53] Campbell S, Roland M, Buetow S (2000). Defining quality of care. Soc Sci Med.

[54] Yang H, Shi L, Lebrun LA, Zhou X, Liu J, Wang H (2013). Development of the Chinese primary care assessment tool: data quality and measurement properties. Int J Qual Health Care.

[55] Wang J, Mo YZ, Bian RW (2013). Evaluation of reliability and validity of application of the Chinese version of 8-item Morisky medication adherence scale in patients with type 2 diabetes [in Chinese]. Chin J Diabetes.

[56] Zhang XX, Wu SY, Wang FS, Mayinuer YSF, Sun KG, Hu K (2017). Association between social support and self-management behaviors among patients with diabetes in community [in Chinese]. J Peking Univ.

[57] Fu AD, Lu GM, Yang J, Liu Q (2011). Application and effect evaluation of clinical pathways for health education in diabetic patients [in Chinese]. Chi Gen Prac.

[58] Fitzgerald JT, Funnell MM, Anderson RM, Nwankwo R, Stansfield RB, Piatt GA (2016). Validation of the revised brief diabetes knowledge test (DKT2). Diabetes Educ.

[59] Clifford S, Pereznieves M, Skalicky A, Reaney M, Coyne KS (2014). A systematic literature review of methodologies used to assess medication adherence in patients with diabetes. Curr Med Res Opin.

[60] Toobert DJ, Hampson SE, Glasgow RE (2000). The summary of diabetes self-care activities measure: results from 7 studies and a revised scale. Diabetes Care.

[61] Saisho Y (2018). Use of diabetes treatment satisfaction questionnaire in diabetes care: importance of patient-reported outcomes. Int J Environ Res Public Health.

[62] Yue Z, Quan C, Xin Y, Juan L, Hongpu H (2016). Application status of information system functions in community health service settings and township health centers in China [in Chinese]. Chi Gen Prac.

[63] Brown JS, Holmes JH, Shah K, Hall K, Lazarus R, Platt R (2010). Distributed health data networks: a practical and preferred approach to multi-institutional evaluations of comparative effectiveness, safety, and quality of care. Med Care.

[64] Wu LY, Xie JF (2019). Survey on knowledge, attitude and behavior in patients with chronic kidney disease [in Chinese]. Chin J Gen Pract.

[65] Kaplan SH, Griffith JL, Price LL, Pawlson LG, Greenfield S (2009). Improving the reliability of physician performance assessment: identifying the “physician effect” on quality and creating composite measures. Med Care.

[66] Scholle SH, Roski J, Adams JL, Dunn DL, Kerr EA, Dugan DP (2008). Benchmarking physician performance: reliability of individual and composite measures. Am J Manag Care.

[67] Lovaglio PG. Benchmarking strategies for measuring the quality of healthcare: problems and prospects. Sci World J. 2012;606154. 10.1100/2012/606154 PMC3361319 22666140

[68] Barclay M, Dixonwoods M, Lyratzopoulos G (2019). The problem with composite indicators. BMJ Qual Saf.

