# Supplementary material for: Development of type 2 diabetes mellitus quality indicators in general practice by a modified Delphi method in Beijing, China
Source: BMC Fam Pract. 2020 Jul 19;21:146. doi: 10.1186/s12875-020-01215-9 (PMC7370510; doi:10.1186/s12875-020-01215-9)
Supplement: Supplementary file 1 — Additional file 1. Rating form in the first round-translation [file 12875_2020_1215_MOESM1_ESM.pdf]

Rating form in the first round of Delphi Survey

Instructions on rating

Importance is defined as the extent to which the indicator is considered important for providing high quality T2DM care in general practice.

Rating of "9" denotes extremely important, "1" denotes extremely unimportant.

Feasibility is defined as the extent to which the indicator could be feasibly used in the general practice setting.

Rating of "9" denotes extremely feasible, "1" denotes extremely unfeasible.

Please put a " √ " on the rating you give for each indicator.

Rating form in the first round

Indicators	Description of indicator	Importance	Feasibility	Comments
**1. Access**				
1.1 Personal doctor	GP is the personal doctor providing continuous care for T2DM patient	1 2 3 4 5 6 7 8 9	1 2 3 4 5 6 7 8 9	
1.2 GP Team	Patient is being managed by a functioning GP team (including a GP, nurse, preventive care physician)	1 2 3 4 5 6 7 8 9	1 2 3 4 5 6 7 8 9	
1.3 Waiting time	Waiting time is reasonable for the patient	1 2 3 4 5 6 7 8 9	1 2 3 4 5 6 7 8 9	
1.4 Health advice	Seeking health advice from the GP team is convenient for the patient	1 2 3 4 5 6 7 8 9	1 2 3 4 5 6 7 8 9	
1.5 Referral access	Patient has ensured referral access to necessary specialist care	1 2 3 4 5 6 7 8 9	1 2 3 4 5 6 7 8 9	

Indicators	Description of indicator	Importance	Feasibility	Comments
**2. Monitoring**				
2.1 Regular follow up	At least 4 times of follow up in the audit year by the GP team	1 2 3 4 5 6 7 8 9	1 2 3 4 5 6 7 8 9	
2.2 Plasma blood glucose monitoring	At least 4 measurements of plasma blood glucose test (fasting or post-prandial) by the GP team in the audit year	1 2 3 4 5 6 7 8 9	1 2 3 4 5 6 7 8 9	
2.3 HbA1c monitoring	At least 2 measurements of HbA1c test in the audit year	1 2 3 4 5 6 7 8 9	1 2 3 4 5 6 7 8 9	
2.4 BP monitoring	At least 4 measurements of BP by the GP team in the audit year	1 2 3 4 5 6 7 8 9	1 2 3 4 5 6 7 8 9	
2.5 Lipid monitoring	At least 1 measurement of lipid test in the audit year	1 2 3 4 5 6 7 8 9	1 2 3 4 5 6 7 8 9	
2.6 BMI monitoring	At least 4 measurements of BMI in the audit year	1 2 3 4 5 6 7 8 9	1 2 3 4 5 6 7 8 9	
2.7 ECG monitoring	At least 1 measurement of ECG in the audit year	1 2 3 4 5 6 7 8 9	1 2 3 4 5 6 7 8 9	
2.8 Nephropathy monitoring	At least 1 nephropathy examination in the audit year	1 2 3 4 5 6 7 8 9	1 2 3 4 5 6 7 8 9	
2.9 Retinopathy monitoring	At least 1 retinopathy examination in the audit year	1 2 3 4 5 6 7 8 9	1 2 3 4 5 6 7 8 9	
2.10 Neuropathy monitoring	At least 1 peripheral neuropathy examination in the audit year	1 2 3 4 5 6 7 8 9	1 2 3 4 5 6 7 8 9	
2.11 Foot monitoring	At least 1 diabetic foot examination in the audit year	1 2 3 4 5 6 7 8 9	1 2 3 4 5 6 7 8 9	
2.12 Ankle-brachial index monitoring	At least 1 ankle-brachial index examination in the audit year	1 2 3 4 5 6 7 8 9	1 2 3 4 5 6 7 8 9	

Indicators	Description of indicator	Importance	Feasibility	Comments
**3. Health counseling**				
3.1 Diet counseling	Diet counseling is provided for the patient in the audit year	1 2 3 4 5 6 7 8 9	1 2 3 4 5 6 7 8 9	
3.2 Exercise counseling	Exercise counseling is provided for the patient in the audit year	1 2 3 4 5 6 7 8 9	1 2 3 4 5 6 7 8 9	
3.3 Smoking counseling	Smoking counseling is provided for the patient when necessary in the audit year	1 2 3 4 5 6 7 8 9	1 2 3 4 5 6 7 8 9	
3.4 Psychological counseling	Psychological counseling is provided for the patient when necessary in the audit year	1 2 3 4 5 6 7 8 9	1 2 3 4 5 6 7 8 9	
**4. Treatment**				
4.1 Rational use of medicines	(1) Metformin medication in the treatment plan unless contradicted<br>(2) ACEI or ARB medication for patient with hypertension unless contradicted<br>(2) Aspirin medication for patient with ASCVD unless contradicted	1 2 3 4 5 6 7 8 9	1 2 3 4 5 6 7 8 9	
**5. Patient safety**				
5.1 Hypoglycemia awareness counseling	Hypoglycemia awareness counseling is provided for the patient in the audit year	1 2 3 4 5 6 7 8 9	1 2 3 4 5 6 7 8 9	
5.2 Medication safety counseling	Medication safety counseling is provided for the patient in the audit year	1 2 3 4 5 6 7 8 9	1 2 3 4 5 6 7 8 9	
5.3 Emergency help counseling	Emergency help counseling is provided for the patient in the audit year	1 2 3 4 5 6 7 8 9	1 2 3 4 5 6 7 8 9	

Indicators	Description of indicator	Importance	Feasibility	Comments
**6. Records**				
6.1 Follow up records	Follow up records are kept in the audit year	1 2 3 4 5 6 7 8 9	1 2 3 4 5 6 7 8 9	
6.2 Annual management report	Annual management report is kept in the audit year	1 2 3 4 5 6 7 8 9	1 2 3 4 5 6 7 8 9	
6.3 Physical examination report	Annual physical examination report is kept in the audit year	1 2 3 4 5 6 7 8 9	1 2 3 4 5 6 7 8 9	
**7. Health status**				
7.1 Blood glucose target	The patient's latest fasting blood glucose is between 4.4-7.0 mmol/L; and post prandial blood glucose <10.0 mmol/L in the audit year	1 2 3 4 5 6 7 8 9	1 2 3 4 5 6 7 8 9	
7.2 HbA1c target	The patient's latest HbA1c <7%;<br>or HbA1c<8% (for patient with severe hypoglycemia or age≥80 or micro or macro vascular complications) in the audit year	1 2 3 4 5 6 7 8 9	1 2 3 4 5 6 7 8 9	
7.3 BP target	The patient's latest BP <130/80 mmHg;<br>or BP <140/90 mmHg for old patient or patient with CHD in the audit year	1 2 3 4 5 6 7 8 9	1 2 3 4 5 6 7 8 9	
7.4 Blood lipid target	The patient's latest LDL-C<1.8 mmol/L (with ASCVD);<br>or LDL-C<2.6 mmol/L (without ASCVD) in the audit year	1 2 3 4 5 6 7 8 9	1 2 3 4 5 6 7 8 9	
7.5 BMI target	The patient's latest BMI<24 kg/m2 in the audit year	1 2 3 4 5 6 7 8 9	1 2 3 4 5 6 7 8 9	
7.6 Hypoglycemia episodes	The patient's episodes of hypoglycemia (including symptomatic hypoglycemia and test results) in the audit year	1 2 3 4 5 6 7 8 9	1 2 3 4 5 6 7 8 9	
7.7 Incidence of complications	Incidence of complications in the audit year	1 2 3 4 5 6 7 8 9	1 2 3 4 5 6 7 8 9	
7.8 Quality of life	Quality of life of patient in the audit year	1 2 3 4 5 6 7 8 9	1 2 3 4 5 6 7 8 9	

Indicators	Description of indicator	Importance	Feasibility	Comments
7.9 T2DM related admissions to hospital	The patient's T2DM related admissions to hospital in the audit year	1 2 3 4 5 6 7 8 9	1 2 3 4 5 6 7 8 9	
7.10 T2DM related admission days in hospital	The patient's T2DM related admission days in hospital in the audit year	1 2 3 4 5 6 7 8 9	1 2 3 4 5 6 7 8 9	
**8. Patient experience**				
8.1 Satisfaction with treatment	The patient's perceived satisfaction with treatment in the audit year	1 2 3 4 5 6 7 8 9	1 2 3 4 5 6 7 8 9	
8.2 Satisfaction with communication	The patient's perceived satisfaction with communication in the audit year	1 2 3 4 5 6 7 8 9	1 2 3 4 5 6 7 8 9	
8.3 Family orientation	The GP team provide care with family orientation for the patient	1 2 3 4 5 6 7 8 9	1 2 3 4 5 6 7 8 9	
**9. Self-management**				
9.1 Adherence to medication	The patient's adherence to medication in the audit year	1 2 3 4 5 6 7 8 9	1 2 3 4 5 6 7 8 9	
9.2 Adherence to healthy behavior	The patient's adherence to healthy behavior in the audit year	1 2 3 4 5 6 7 8 9	1 2 3 4 5 6 7 8 9	

Abbreviations: GP: general practitioner; T2DM: type 2 diabetes mellitus; HbA1c: glycosylated hemoglobin; BP: blood pressure; BMI: body mass index; ECG: electrocardiogram; ACEI: angiotensin converting enzyme inhibitor ; ARB: angiotensin receptor blocker; ASCVD: arteriosclerotic cardiovascular disease; CHD: coronary heart disease; LDL-C: low density lipoprotein cholesterol.

Recommendation of additional indicators and further comments

Recommendation of additional indicators	Description of the indicator	Reasons
Further comments		
