# Supplementary material for: Development of type 2 diabetes mellitus quality indicators in general practice by a modified Delphi method in Beijing, China
Source: BMC Fam Pract. 2020 Jul 19;21:146. doi: 10.1186/s12875-020-01215-9 (PMC7370510; doi:10.1186/s12875-020-01215-9)
Supplement: Supplementary file 2 — Additional file 2. Results of ratings in the Delphi survey and consensus meeting [file 12875_2020_1215_MOESM2_ESM.pdf]

Results of ratings in the Delphi survey and consensus meeting

Table 7 Rating distribution in the first round (n=30)

Indicators	Importance					Feasibility				
	Median	1~3	4~6	7~9	Percentage in 7~9	Median	1~3	4~6	7~9	Percentage in 7~9
1. Access										
1.1 Personal doctor	9.0	0	1	29	96.7%	8.0	0	3	27	90.0%
1.2 GP Team	9.0	0	1	29	96.7%	8.0	0	4	26	86.7%
1.3 Waiting time	9.0	0	1	29	96.7%	8.0	0	1	29	96.7%
1.4 Health advice	8.0	2	2	26	86.7%	7.0	1	8	21	70.0%
1.5 Referral access	9.0	1	1	28	93.3%	8.0	0	5	25	83.3%
2. Monitoring										
2.1 Regular follow up	9.0	0	2	28	93.3%	9.0	0	2	28	93.3%
2.2 Plasma blood glucose monitoring	9.0	0	0	30	100.0%	9.0	0	2	28	93.3%
2.3 HbA1c monitoring	9.0	0	1	29	96.7%	7.5	0	8	22	73.3%
2.4 BP monitoring	9.0	0	0	30	100.0%	9.0	0	1	29	96.7%
2.5 Lipid monitoring	9.0	0	0	30	100.0%	9.0	0	3	27	90.0%
2.6 BMI monitoring	9.0	0	1	29	96.7%	9.0	0	4	26	86.7%
2.7 ECG monitoring	9.0	0	2	27	90.0%	9.0	0	3	27	90.0%
2.8 Nephropathy monitoring	9.0	0	0	30	100.0%	8.0	0	5	25	83.3%
2.9 Retinopathy monitoring	9.0	0	0	30	100.0%	7.0	3	7	20	66.7%
2.10 Neuropathy monitoring	9.0	0	1	29	96.7%	8.0	4	3	23	76.7%
2.11 Foot monitoring	9.0	0	1	29	96.7%	8.0	0	6	24	80.0%
2.12 Ankle-brachial index monitoring	9.0	1	2	27	90.0%	7.0	1	10	19	63.3%

3. Health counseling									
3.1 Diet counseling	9.0	0	1	29	96.7%	8.5	0	5	25	83.3%
3.2 Exercise counseling	9.0	0	1	29	96.7%	8.5	0	6	24	80.0%
3.3 Smoking counseling	9.0	0	1	29	96.7%	9.0	0	8	22	73.3%
3.4 Psychological counseling	9.0	0	2	28	93.3%	7.0	3	6	21	70.0%
4. Treatment										
4.1 Rational use of medicines	9.0	0	1	29	96.7%	8.0	1	4	25	83.3%
5. Patient safety										
5.1 Hypoglycemia awareness counseling	9.0	0	0	30	100.0%	9.0	0	3	27	90.0%
5.2 Medication safety counseling	9.0	0	0	30	100.0%	8.0	0	2	28	83.3%
5.3 Emergency help counseling	9.0	1	1	28	93.3%	8.0	2	5	23	76.7%
6. Records										
6.1 Follow up records	9.0	0	2	28	93.3%	9.0	1	2	27	90.0%
6.2 Annual management report	9.0	1	1	28	93.3%	9.0	2	1	27	90.0%
6.3 Physical examination report	9.0	1	1	28	93.3%	9.0	2	1	27	90.0%
7. Health status										
7.1 Blood glucose target	9.0	0	0	30	100.0%	8.5	0	5	25	83.3%
7.2 HbA1c target	9.0	0	0	30	100.0%	8.0	0	8	22	73.3%
7.3 BP target	9.0	0	0	30	100.0%	8.0	0	7	23	76.7%
7.4 Blood lipid target	9.0	0	0	30	100.0%	8.0	0	7	23	76.7%
7.5 BMI target	9.0	0	1	29	96.7%	8.0	0	7	23	76.7%
7.6 Hypoglycemia episodes	9.0	0	1	28	93.3%	8.0	2	5	22	73.3%
7.7 Incidence of Complications	9.0	0	0	30	100.0%	8.0	1	8	21	70.0%
7.8 Quality of life	9.0	0	2	28	93.3%	7.0	1	8	21	70.0%
7.9 T2DM related admissions to hospital	9.0	0	2	28	93.3%	8.0	2	6	22	73.3%
7.10 T2DM related admission days in hospital	8.0	2	3	25	83.3%	8.0	3	7	20	66.7%

8. Patient experience										
8.1 Satisfaction with treatment	9.0	0	1	29	96.7%	8.5	0	3	27	90.0%
8.2 Satisfaction with communication	9.0	0	1	29	96.7%	8.0	0	4	26	86.7%
8.3 Family orientation	8.5	0	3	27	90.0%	7.0	1	4	25	83.3%
9. Self-management										
9.1 Adherence to medication	9.0	0	1	29	96.7%	8.0	0	3	27	90.0%
9.2 Adherence to healthy behavior	9.0	0	3	27	90.0%	7.0	1	5	24	80.0%

Abbreviations: GP: general practitioner; HbA1c: glycosylated hemoglobin; BP: blood pressure; BMI: body mass index; ECG: electrocardiogram; T2DM: type 2 diabetes mellitus.

Table 8 Rating distribution in the second round (n=27)

Indicators	Importance					Feasibility				
	Median	1~3	4~6	7~9	Percentage in 7~9	Median	1~3	4~6	7~9	Percentage in 7~9
1. Access										
1.1 Personal doctor	9.0	0	0	27	100.0%	9.0	0	1	26	96.3%
1.2 GP Team	9.0	0	1	26	96.3%	8.0	0	3	24	88.9%
1.3 Waiting time	9.0	0	1	26	96.3%	8.0	0	2	25	92.6%
1.4 Health advice	9.0	0	3	24	88.9%	8.0	0	4	23	85.2%
1.5 Referral access	9.0	0	4	23	85.2%	8.0	0	4	23	85.2%
2. Monitoring										
2.1 Regular follow up	9.0	0	1	26	96.3%	9.0	0	0	27	100.0%
2.2 Plasma blood glucose monitoring	9.0	0	1	26	96.3%	9.0	0	3	24	88.9%
2.3 HbA1c monitoring	9.0	0	0	27	100.0%	8.0	0	4	23	85.2%
2.4 BP monitoring	9.0	0	1	26	96.3%	9.0	0	1	26	96.3%
2.5 Lipid monitoring	9.0	0	0	27	100.0%	8.0	0	1	26	96.3%
2.6 BMI monitoring	9.0	0	0	27	100.0%	9.0	0	2	25	92.6%
2.7 Waist circumference monitoring	9.0	0	0	27	100.0%	9.0	0	2	25	92.6%
2.8 ECG monitoring	9.0	0	1	26	96.3%	8.0	0	3	24	88.9%
2.9 Nephropathy monitoring	9.0	0	0	27	100.0%	8.0	0	3	24	88.9%
2.10 Retinopathy monitoring	9.0	0	1	26	96.3%	8.0	0	7	20	74.1%
2.11 Neuropathy monitoring	9.0	0	1	26	96.3%	8.0	1	6	20	74.1%
2.12 Foot monitoring	9.0	0	0	27	100.0%	8.0	0	4	23	85.2%
3. Health counseling										
3.1 Diet counseling	9.0	0	0	27	100.0%	8.0	0	1	26	96.3%
3.2 Exercise counseling	9.0	0	0	27	100.0%	8.0	0	2	25	92.6%

3.3 Smoking counseling	9.0	0	1	26	96.3%	8.0	1	6	20	74.1%
3.4 Psychological counseling	9.0	0	0	27	100.0%	7.0	0	11	16	59.3%
3.5 Hypoglycemia awareness counseling	9.0	0	0	27	100.0%	9.0	0	3	24	88.9%
3.6 Medication safety counseling	9.0	0	0	27	100.0%	8.0	0	5	22	81.5%
3.7 Emergency help counseling	9.0	0	0	27	100.0%	8.0	0	3	24	88.9%
4. Records										
4.1 Follow up records	9.0	0	0	27	100.0%	9.0	0	1	26	96.3%
4.2 Annual management summary report	9.0	0	0	27	100.0%	9.0	0	0	27	100.0%
5. Health status										
5.1 Blood glucose target	9.0	1	1	25	92.6%	8.0	0	3	24	88.9%
5.2 HbA1c target	9.0	0	1	26	96.3%	8.0	0	5	22	81.5%
5.3 BP target	9.0	0	1	26	96.3%	9.0	0	3	24	88.9%
5.4 Blood lipid target	9.0	0	1	26	96.3%	8.0	0	6	21	77.8%
5.5 BMI target	9.0	0	1	26	96.3%	8.0	1	6	20	74.1%
5.6 Quality of life	8.0	1	2	24	88.9%	7.0	0	9	18	66.7%
6. Patient satisfaction										
6.1 Satisfaction with treatment	9.0	1	0	26	96.3%	8.0	0	1	26	96.3%
6.2 Satisfaction with communication	8.5	0	0	26	96.3%	8.0	0	4	22	81.5%
7. Self-management										
7.1 Knowledge of self-management	9.0	0	1	26	96.3%	8.0	0	6	21	77.8%
7.2 Adherence to medication	9.0	0	0	27	100.0%	8.0	0	2	25	92.6%
7.3 Adherence to healthy behavior	9.0	0	1	26	96.3%	7.0	0	7	20	74.1%

Abbreviations: GP: general practitioner; HbA1c: glycosylated hemoglobin; BP: blood pressure; BMI: body mass index; ECG: electrocardiogram.

Table 9 Rating distribution in the consensus meeting (n=9)

Indicators	Importance					Feasibility				
	Median	1~3	4~6	7~9	Percentage in 7~9	Median	1~3	4~6	7~9	Percentage in 7~9
1. Access										
1.1 Personal doctor	9.0	0	0	9	100.0%	9.0	0	0	9	100.0%
1.2 GP team	9.0	0	0	9	100.0%	9.0	0	0	9	100.0%
1.3 Waiting time	9.0	0	0	9	100.0%	9.0	0	0	9	100.0%
1.4 Health advice	9.0	0	0	9	100.0%	9.0	0	0	9	100.0%
1.5 Referral access	9.0	0	0	9	100.0%	9.0	0	0	9	100.0%
2. Monitoring										
2.1 Regular follow up	9.0	0	0	9	100.0%	9.0	0	0	9	100.0%
2.2 Plasma blood glucose monitoring	9.0	0	0	9	100.0%	9.0	0	0	9	100.0%
2.3 HbA1c monitoring	9.0	0	0	9	100.0%	9.0	0	0	9	100.0%
2.4 BP monitoring	9.0	0	0	9	100.0%	9.0	0	0	9	100.0%
2.5 Lipid monitoring	9.0	0	0	9	100.0%	9.0	0	0	9	100.0%
2.6 BMI monitoring	9.0	0	0	9	100.0%	9.0	0	0	9	100.0%
2.7 Waist circumference monitoring	9.0	0	0	9	100.0%	9.0	0	0	9	100.0%
2.8 ECG monitoring	9.0	0	0	9	100.0%	9.0	0	0	9	100.0%
2.9 Nephropathy monitoring	9.0	0	0	9	100.0%	9.0	0	0	9	100.0%
2.10 Retinopathy monitoring	9.0	0	0	9	100.0%	8.0	0	0	9	100.0%
2.11 Peripheral neuropathy monitoring	9.0	0	0	9	100.0%	8.0	0	0	9	100.0%
2.12 Foot monitoring	9.0	0	0	9	100.0%	9.0	0	0	9	100.0%
3. Health counseling										
3.1 Diet counseling	9.0	0	0	9	100.0%	9.0	0	0	9	100.0%
3.2 Exercise counseling	9.0	0	0	9	100.0%	9.0	0	0	9	100.0%

3.3 Psychological assessment or counseling	9.0	0	1	8	88.9%	7.0	0	2	7	77.8%
3.4 Smoking assessment or counseling	9.0	0	0	9	100.0%	9.0	0	0	9	100.0%
3.5 Hypoglycemia awareness counseling	9.0	0	0	9	100.0%	9.0	0	0	9	100.0%
3.6 Medication safety counseling	9.0	0	0	9	100.0%	8.0	0	0	9	100.0%
3.7 Emergency help counseling	9.0	0	0	9	100.0%	8.0	0	0	9	100.0%
4. Records										
4.1 Follow up records	9.0	0	0	9	100.0%	9.0	0	0	9	100.0%
4.2 Annual management summary report	9.0	0	0	9	100.0%	9.0	0	0	9	100.0%
5. Health status										
5.1 Blood glucose target	9.0	0	0	9	100.0%	9.0	0	0	9	100.0%
5.2 HbA1c target	9.0	0	0	9	100.0%	8.0	0	0	9	100.0%
5.3 BP target	9.0	0	0	9	100.0%	9.0	0	0	9	100.0%
5.4 Blood lipid target	9.0	0	0	9	100.0%	9.0	0	0	9	100.0%
5.5 BMI target	9.0	0	0	9	100.0%	8.0	0	2	7	77.8%
5.6 Hypoglycemia episodes	9.0	0	0	9	100.0%	8.0	0	0	9	100.0%
5.7 T2DM related admissions to hospital	9.0	0	0	9	100.0%	8.0	0	2	7	77.8%
6. Patient satisfaction										
6.1 Satisfaction with treatment	8.0	0	0	9	100.0%	8.0	0	0	9	100.0%
6.2 Satisfaction with communication	8.0	0	0	9	100.0%	8.0	0	0	9	100.0%
7. Self-management										
7.1 Knowledge of self-management	9.0	0	0	9	100.0%	8.0	0	0	9	100.0%
7.2 Adherence to medication	9.0	0	0	9	100.0%	8.0	0	0	9	100.0%
7.3 Adherence to healthy behavior	9.0	0	0	9	100.0%	8.0	0	0	9	100.0%

Abbreviations: GP: general practitioner; HbA1c: glycosylated hemoglobin; BP: blood pressure; BMI: body mass index; ECG: electrocardiogram; T2DM: type 2 diabetes mellitus.
